# Trions Stimulate Electronic Coupling in Colloidal Quantum Dot Molecules

**DOI:** 10.1021/acs.chemmater.4c02809

**Published:** 2024-11-22

**Authors:** Jordi Llusar, Juan I. Climente

**Affiliations:** † 518636 BCMaterials, Basque Center for Materials, Applications, and Nanostructures, E-48940 Leioa, Spain; ‡ Departament de Química Física i Analítica, 16748 Universitat Jaume I, E-12080 Castelló de la Plana, Spain

## Abstract

Recent synthetic progress has enabled the controlled fusion of colloidal CdSe/CdS quantum dots in order to form dimers, manifesting electronic coupling in their optical response. While this “artificial H_2_ molecule” constitutes a milestone toward the development of nanocrystal chemistry, the strength of the coupling has proven to be smaller than intended. The reason is that, when an exciton is photoinduced in the system, the hole localizes inside the CdSe cores and captures the electron, often preventing substantial delocalization across the dimer. Here, we predict, by means of k·p theory and configuration interaction calculations, that using trions instead of neutral excitons or biexcitons restores the electron delocalization. Positive trions are particularly apt because the strong hole–hole repulsion makes electron delocalization robust against moderate asymmetries in the cores, thus maintaining a homodimer-like behavior. The hybridization energies are sufficiently large to preserve the molecular character beyond cryogenic temperatures.

## Graphical abstract

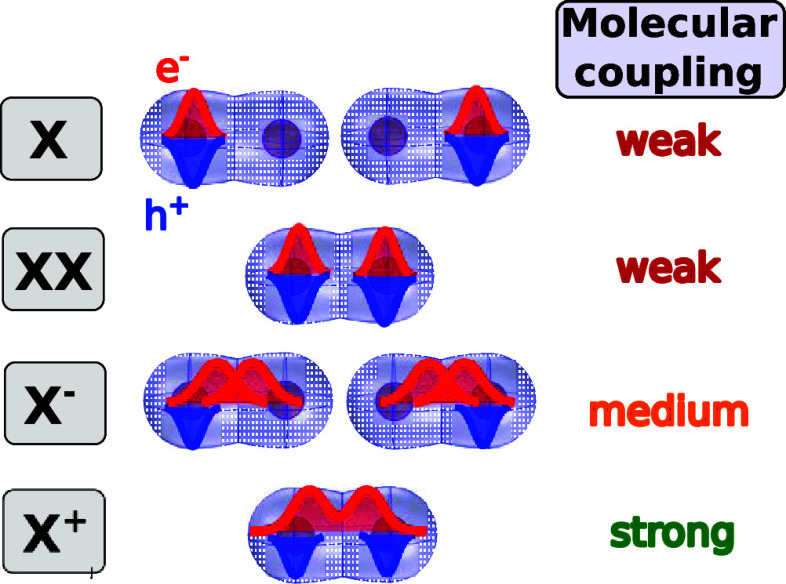

## Introduction

The discrete electronic structure of semiconductor quantum dots (QDs) makes them reminiscent of atoms.^1,2^ It is then natural to pursue the formation of “artificial molecules” by coupling QDs in such a way that their optoelectronic properties differ from those of the individual components. A key magnitude to this end is the strength of electron or hole tunnel coupling between neighboring QDs, which is the solid-state analogue of the chemical bond in molecules.^3–5^ Quantum dot molecules (QDMs) were successfully built in all-solid systems, where electronic coupling and orbital hybridization were confirmed in pairs of vertically^6–8^ or laterally^9,10^ coupled QDs. The charge and spin entanglement associated with the molecular bond was exploited to demonstrate different qubit operations.^11–16^ Tunneling energies, however, were in the range of μeV to few meV. This implied the need for cryogenic temperatures and fragility to system nonidealities breaking the energy resonance between QDs, such as size dispersion and misalignment.^9,17,18^

Colloidal QDs offer an alternative platform to produce QDMs, with larger confinement energies—which have the potential to preserve quantum behavior at high temperatures, access to wet-chemical manipulation, and lower cost of production than all-solid QDMs. Early attempts to synthesize colloidal QDMs were restricted by the use of long and high interdot barriers (as in DNA-connected QDs or CdSe/CdS dumbbells) or by pronounced asymmetries of the constituents (as in CdSe tetrapods or CdSe/CdS dot-in-rod heterostructures).^19–21^ The former factor quenched electronic tunneling, which decays exponentially with the barrier length. The latter gave the molecules a strongly heteronuclear character such that the spectrum was formed by perturbed states of the individual atoms rather than by truly covalent bonds shared between them.

Remarkable progress in the growth of colloidal QDMs has taken place in the past few years. By combining small—yet highly monodisperse—CdSe cores, passivating them with thin CdS shells, and fusing them in pairs, Cui and co-workers succeeded in producing peanut-shaped homodimers whose optical properties clearly differed from those of the fused monomers.^22,23^ The differences included a red-shifted onset of absorption and photoluminescence,^22^ enhancement of the polarization along the molecular axis,^24^ and reduced Auger recombination.^25,26^ Subsequent synthetic refinements have enabled precise control of the CdS neck width^27^ and core-to-core distance.^28^

The electronic structure of CdSe/CdS QDMs is designed so as to maximize electron tunnel coupling.^28,29^ The use of small CdSe cores (radii ∼1.4 nm), along with the low CdSe/CdS conduction band offset (∼0.1 eV), favors electron delocalization into the shell. Because the CdS shell is kept thin (∼1–2 nm), and the neck is wide, electronic coupling between the two adjacent CdSe cores is expected to be significant. As a matter of fact, at the independent-particle level, the energy splitting between bonding and antibonding molecular orbitals (hybridization energy, Δ_e_) was estimated to be in the range of a few tens of meV, by both effective mass^29^ and atomistic pseudopotential^30^ calculations. However, the electron in these structures is photoinduced, which means that it interacts with a hole to form an exciton. Unlike electrons, holes—which are heavier and experience a high valence band offset—are strongly confined inside the CdSe cores. Since exciton binding energies (stimulated by dielectric confinement) are 1 order of magnitude greater than Δ_e_, Coulomb attraction drives the electron toward the core where the hole lies, thus preventing it from tunneling across the dimer.^22,29^ As a result, theoretical analysis of excitons in colloidal QDMs has shown that much of the redshift observed in the optical spectrum of dimers is not related to electronic coupling, as initially expected.^22^ Rather, it originates in the partial deconfinement of the localized exciton, which upon fusion of two monomers can extend its tail slightly farther into the CdS shell.^30^ QDMs with very thin (∼1 nm) shells may still exhibit substantial exciton hybridization, but these structures are chemically less stable.^28^

An additional problem of state-of-the-art QDMs is that the same conditions that foster electron delocalization, namely, the use of small CdSe cores, make the system very sensitive to small deviations from the homonuclear character. Being in the strong confinement regime, a radius variation of ∼0.1 nm between the two CdSe cores suffices to open a gap between their energy levels exceeding Δ_e_. This suppresses the sharing of excitons or biexcitons between the cores.^26^

In this work, we propose an alternative strategy to overcome the aforementioned difficulties and attain robust electronic coupling in QDMs. The key point is to replace the use of neutral excitons and biexcitons with positively or negatively charged excitons (trions). As we shall see, trions restore electron tunneling by means of the additional Coulomb attraction and repulsion terms, which are missing in excitons. In the case of positive trions, the repulsive terms further provide stability of the electronic bond against moderate core size inhomogeneities. The occasional presence of trions (coexisting with excitons) in QDMs has been experimentally verified, likely enabled by reduced nonradiative Auger rates in these systems.^22,31^ In light of our findings, they do provide substantial electronic coupling to colloidal QDMs. Stimulating their presence in homodimers—for example by means of recently developed electronic gating^32^—should translate into a net increase of the coupling strength. Spectroscopic signatures to confirm the presence of electronic coupling are proposed.

## Theoretical Methods

We calculate electron (*e*), hole (*h*), exciton (*X*), positive trion (*X* ^+^), negative trion (*X* ^–^), and biexciton (*XX*) states in wurtzite CdSe/CdS QDMs using the same model and material parameters as in ref 26. Thus, simulations are carried out within effective mass theory using single-band Hamiltonians for *e* and *h*. The Poisson equation is integrated accounting for the strong dielectric confinement of the QDM, which boosts the strength of the Coulomb interactions. Many-body eigenstates are computed using a full configuration interaction (CI) method. The basis set is formed by all possible combinations of the first 20 independent electron and hole spin–orbitals. It is worth noting that, using our model or similar ones, satisfactory descriptions of the experimental behavior of *X* and *XX* in QDMs have been reported.^22,26,29^ As compared to self-consistent methods used in earlier simulations,^22,29^ CI methods offer the advantage of describing not only the ground state but also low-lying ones. This will be important to compare the hybridization energies of different excitonic species on equal footing. Details of the model and material parameters are given in Section S1 (Supporting Information).

## Results and Discussion

To illustrate and validate our strategy, we calculate *e*, *h*, *X*, *X* ^+^, *X* ^–^, and *XX* states in colloidal QDMs with the geometry shown in Figure 1a. Two spherical CdSe cores, with radii of *r*_l_ and *r*_r_ (the subindexes stand for left and right, respectively), are surrounded by CdS shells. The shells are spherical with radii *R*_l_ and *R*_r_, except in the direction of coupling. Here, shell ripening broadens the neck width.^27^ The resulting geometry is well described by making the shells ellipsoidal, with semimajor axes *n*_l_ = *n*_r_ = *n*.^29^ In homodimers, where *R*_l_ = *R*_r_ = *R*, *n* sets the neck width as $D_{\mathrm{neck}}=2R\sqrt{1-(R/n{)}^{2}}$ . Thus, in the limit of *n* = *R*, the dimer is made of two spheres with a tangential surface contact (*D*_neck_ = 0). When *n* increases, the CdS shell evolves toward rod shape (*D*_neck_ = 2*R*), which maximizes tunnel coupling (a few geometries are represented in Figure S1). In the following, we consider a prototypical QDM with optimal coupling properties, namely, *r*_l_ = *r*_r_ = 1.35 nm, *R*_l_ = *R*_r_ = 3.4 nm (that gives a shell thickness of 2.05 nm), and *n* = 7 nm.^26^ The latter yields *D*_neck_ ≈ 6 nm, i.e., a quasi-rod-like shell. These specific dimensions correspond to the geometry displayed in Figure 1a. Different geometries may change the coupling strength but not the conceptual differences between trions and excitons we aim to evidence.

**Figure 1:**
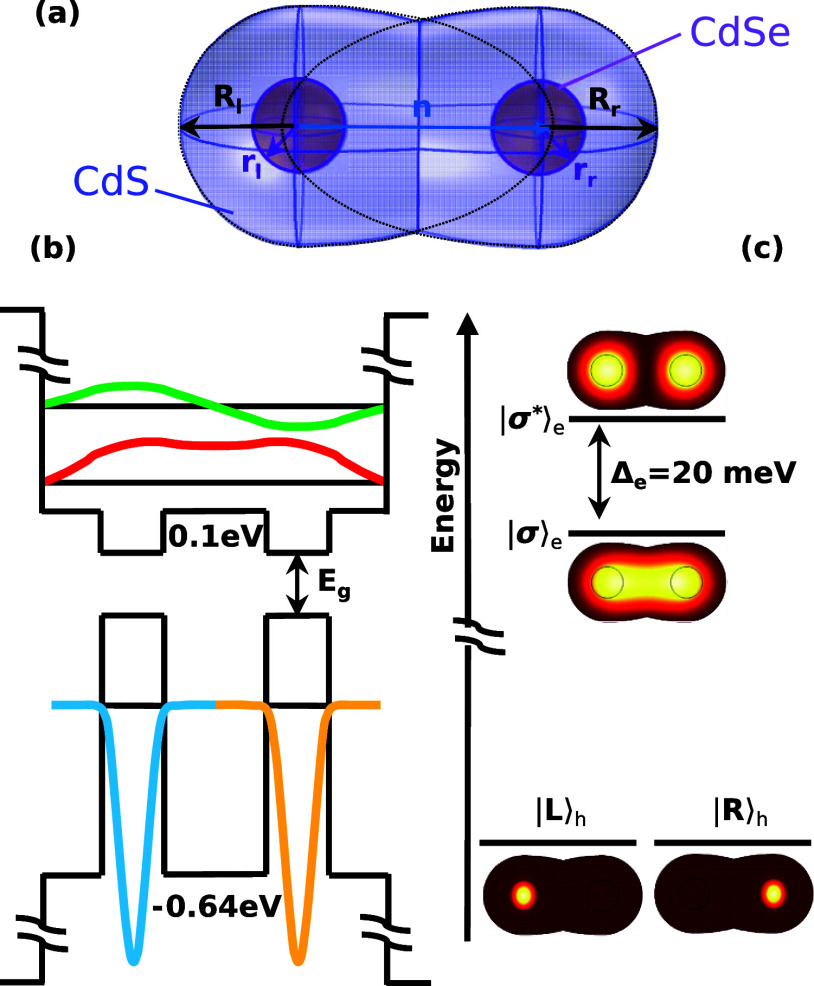
(a) Schematic of the CdSe/CdS QDM geometry. CdSe cores (brownish spheres) are surrounded by CdS shells (blue), which constitute a low barrier for the electron to tunnel between the cores. (b) Confining potential and wave function of the lowest electron (top) and highest hole (bottom) states along the molecular axis. (c) Two-dimensional representation of the normalized electron (top) and hole (bottom) charge densities. The states are calculated in the independent particle approximation. Electrons hybridize to form bonding and antibonding molecular orbitals. Holes stay localized in the CdSe cores.

In the first approach, we study the electronic structure of *e* and *h* within the independent particle picture (no mutual interaction). Figure 1b depicts the confining potential seen by the carriers together with the lowest *e* (top part) and the highest *h* (bottom part) states. The figure shows that electrons take advantage of the low conduction band offset (0.1 eV in our simulations) to delocalize and form bonding (σ) and antibonding (σ*) molecular orbitals. By contrast, holes are strongly confined inside the CdSe cores (either left or right, |*L*⟩_h_ and |*R*⟩_h_, the two *s*-like orbitals being degenerate). This is due to their heavier mass and the high valence band offset (0.64 eV). The same contrasting behavior is observed when plotting *e* and *h* charge densities on the QDM plane (see Figure 1c).

It is worth noting that the energy splitting between the *e* states, Δ_e_ = 20 meV, is at least 1 order of magnitude greater than that in self-assembled and electrostatically defined QDMs.^8,10,12^ In principle, this supports the prospects of persistent molecular coupling at or near room temperature. Unfortunately, the inclusion of *e*–*h* interaction changes this picture drastically. For neutral *X*, the Coulomb potential generated by the localized hole states captures the electron in the vicinity of the CdSe cores. As a result, the exciton ground state shows a low electron charge density (ρ_e_) in the neck region. This is illustrated in the top panel of Figure 2. This observation is consistent with earlier theoretical studies^22,29^ and implies the suppression of electron tunnel coupling.^30^ A similar behavior is observed for *XX* (bottom panel). Here, the ground state is constituted by one *X* in each QD of the homodimer, with very weak (dipole–dipole) interaction between them.^26^

**Figure 2:**
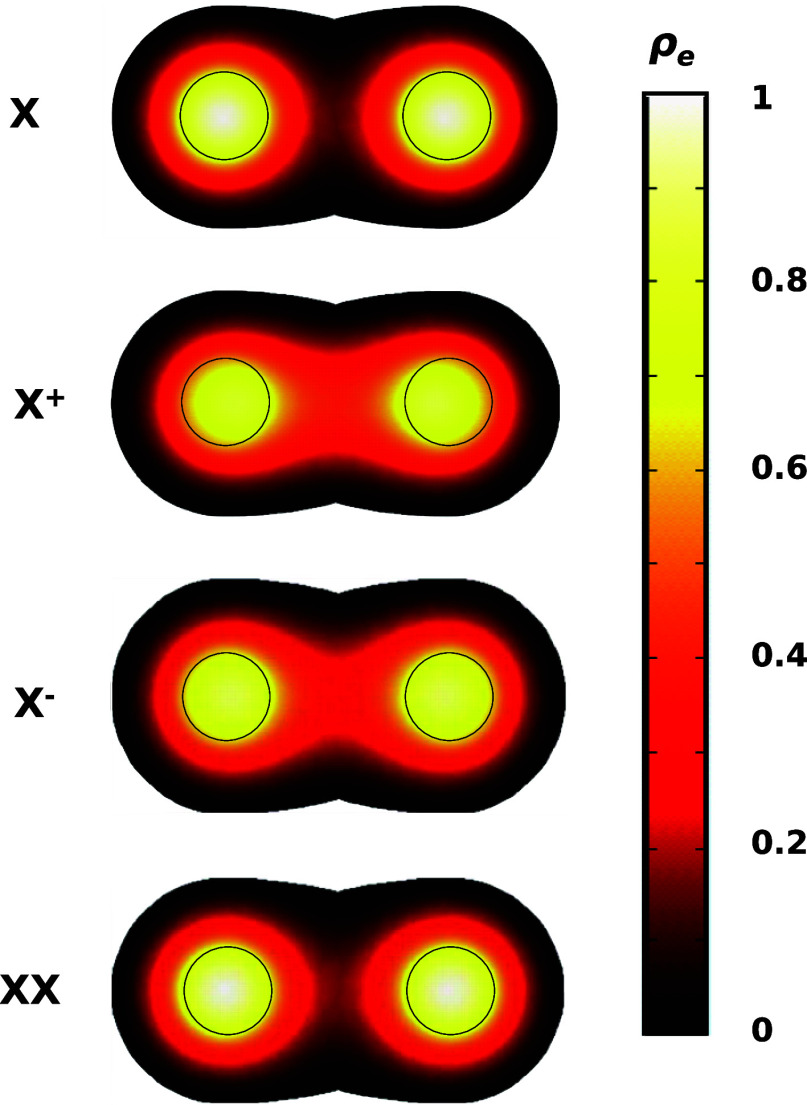
Normalized electron charge density in a typical homodimer for different excitonic complexes. Excitons and biexcitons (top and bottom plots) show suppressed charge density in the neck region, while positive and negative trions (central plots) show restored tunnel coupling.

Interestingly, a completely different behavior is found for positive (*X* ^+^) and negative (*X* ^–^) trions—central panels in Figure 2—which do exhibit substantial electron charge density in the neck, akin to that of noninteracting electrons. The interpretation of this result can be traced back to the extra attractive and repulsive Coulomb terms in trions, which have a direct impact on the hybridization energy.^12^ *X* ^–^ can be seen as an electron interacting with an exciton. The *e*–*X* binding energy is much smaller than the *e*–*h* one within *X* because it is a charge–dipole interaction as opposed to a charge–charge one. Consequently, the extra electron is more free to tunnel between the QDs. *X* ^+^, in turn, can be seen as an analogue of a H_2_^+^ molecule, where the electron is covalently shared by two attractive holes, whose position is fixed.

The stronger electronic coupling of trions observed in Figure 2 holds for different QDM geometries. In Figure S3 (Section S2), we consider QDMs with different shell thicknesses. Exciton coupling increases as the shell becomes thinner, and it becomes significant for the thinnest shells (*R* = 2.4 nm, corresponding to a CdS thickness of 1.05 nm), which is in agreement with atomistic studies.^28^ Yet, in all instances, the coupling becomes stronger when a trion is used instead.

To explore if the electronic coupling of trion states can withstand high temperatures, we need to go beyond the ground state and study the energy splitting and molecular character of the low-lying excited states as well. This is done in Figure 3a, which shows the energy levels of the lowest *X*, *X* ^+^, *X* ^–^, and *XX* states, along with the main CI configuration of each state (schematic insets). We find that most *X* ^+^ and *X* ^–^ states have a main configuration with net bonding (σ) or antibonding (σ*) electron character. By contrast, *X* and *XX* states are nonbonding (the main configuration has comparable weight of σ and σ* orbitals). The latter closely corresponds to localized orbitals. For this reason, in the schematics of Figure 3a, we use delocalized orbitals (|σ⟩_e_, |σ*⟩_e_) for trions but localized ones (|*L*⟩_e_, |*R*⟩_e_) for *X* and *XX*. Details about the rotation between basis sets are given in Section S3 of the SI.

**Figure 3:**
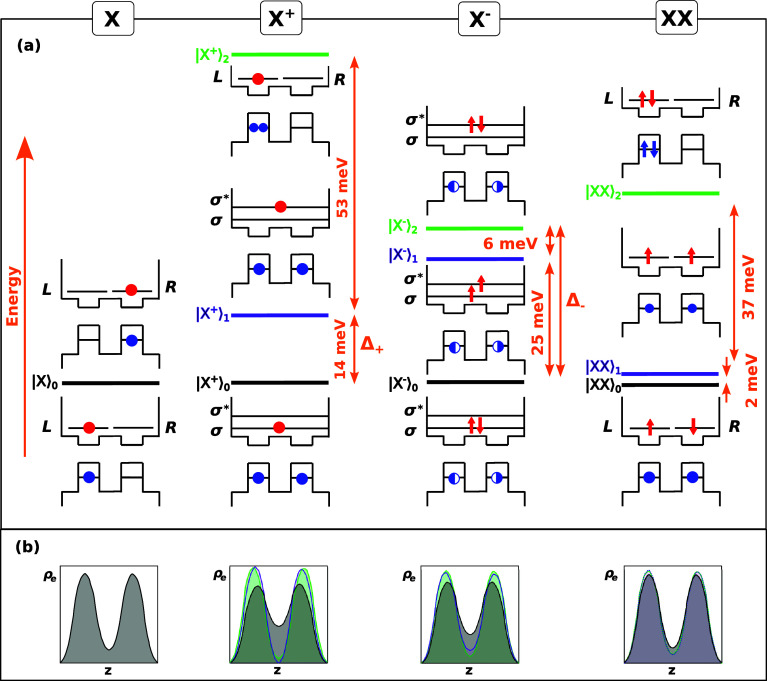
(a) Energy levels of the QDM for different excitonic complexes. The schematic insets show the dominant electronic configurations in the CI expansions for each state. Red and blue circles stand for electrons and holes, respectively. Arrows are used to denote the spin when relevant. Blue semicircles indicate 50% probability of finding the hole in a given QD, i.e., $|h\rangle =(|L\rangle_{h}\pm |R\rangle_{h})/\sqrt{2}$ . (b) Cross-section of the normalized electron charge density along the coupling axis of the QDM for all of the states under study. The line colors correspond to those used in panel (a). Trion ground states show a significant bonding character, with sizable hybridization energies (Δ_+_, Δ_–_).

In the case of *X*, the left-most panel in Figure 3a, the ground state (|*X*⟩_0_) is defined by two orbitals parts of nearly identical energy, which can be approximated as |*X*_L_⟩ ≈ |*L*⟩_e_ |*L*⟩_h_ and |*X*_R_⟩ ≈ |*R*⟩_e_ |*R*⟩_h_. These correspond to an *X* localized in the left QD or in the right QD. Indirect *X* states, where *e* and *h* sit in different QDs, miss much of the *e*–*h* attraction felt by |*X*_L_⟩ and |*X*_R_⟩. For this reason, they are higher in energy beyond the scale of Figure 3a. The localized nature of the *X* ground state is confirmed once more in the left-most panel of Figure 3b, which depicts a cross-section of the electron charge density along the QDM axis, averaged for all of the quasi-degenerate states of |*X*⟩_0_. The plot evidences that excitons are mostly localized in the QDs, with scarce presence in the barrier.

A similar situation is found for *XX*, as shown in the right-most panels in Figure 3. There are three low-lying states (|*XX*⟩_0_, |*XX*⟩_1_, and |*XX*⟩_2_) and all of them localize most of the electronic charge in the QDs (see Figure 3b). The explanation can be found by analyzing the electronic configuration. In the lowest states (|*XX*⟩_0_, |*XX*⟩_1_), the orbital part is found to be (|*L*⟩_e1_|*R*⟩_e2_ ± |*R*⟩_e1_|*L*⟩_e2_) (|*L*⟩_h1_|*R*⟩_h2_ ± |*R*⟩_h1_|*L*⟩_h2_), where the ± sign depends on the electron and hole spins (singlet or triplet). These configurations reflect segregated *XX*, with one exciton sitting in each QD. The second excited state (|*XX*⟩_2_), in turn, corresponds to electron and hole spin singlets, with the orbital configuration |*XX*⟩_2_ ≈ (|*L*⟩_e1_|*L*⟩_e2_ + |*R*⟩_e1_|*R*⟩_e2_) (|*L*⟩_h1_|*L*⟩_h2_ + |*R*⟩_h1_|*R*⟩_h2_). This describes an *XX* confined inside one of the two dots of the QDM.

Positive trions, *X* ^+^, differ from the previous species in that electrons display a clear molecular (delocalized) character, as opposed to the atomic (localized) character of *X* and *XX*. As shown in Figure 3a, the ground state (|*X* ^+^⟩_0_) has one hole in each QD—to avoid repulsions—and the electron in a bonding orbital. The corresponding configuration (orbital part) reads $|X^{+}\rangle_{0}\approx |\sigma \rangle_{e}(|L\rangle_{h1}|R\rangle_{h2}\pm |R\rangle_{h1}|L\rangle_{h2})/\sqrt{2}$ , where again ± signs apply for singlet and triplet hole spins, which are quasi-degenerate. The excited state is analogous but with the electron in an antibonding orbital, $|X^{+}\rangle_{1}\approx |\sigma^{*}\rangle_{e}(|L\rangle_{h1}|R\rangle_{h2}\pm |R\rangle_{h1}|L\rangle_{h2})/\sqrt{2}$ . Positioning the electron in the bonding or antibonding orbitals has a direct influence on the electronic density. As can be seen in the *X* ^+^ panel of Figure 3b, |*X* ^+^⟩_0_—gray curve—has a significant presence in the barrier, but |*X* ^+^⟩_1_—purple curve—has a node. The energy splitting between the two states, Δ_X^+^_ = 14 meV, reveals a sizable hybridization energy, unlike that in *X*. By comparison with the independent electron case in Figure 1c, one can notice that Δ_X^+^_ ≠ Δ_e_. The reason is that Δ_X^+^_ is not set by the mechanical electron tunnel coupling only but also by the balance between Coulomb terms.^12^ In *X* ^+^, the electron in the σ orbital benefits from simultaneous attractions with both holes, as in a covalently bonded H_2_^+^ molecule. By contrast, that in the σ* orbital is closer to an ionic bond, with the peaks of electronic density closer to one of the holes but far from the other. The same trend, Δ_X_ ≪ Δ_X^+^_ ≲ Δ_e_, holds for homodimers with any neck width (Section S4 of the SI).

Negative trions, *X* ^–^, present molecular electronic character, as well. Contrary to the *X* case, the hole does not capture electrons inside its QD because electron–electron repulsions prevent it. The resulting ground state—|*X* ^–^⟩_0_, black line—is then given by an orbital configuration $|X^{-}\rangle_{0}\approx |\sigma \rangle_{e1}|\sigma \rangle_{e2}(|L\rangle_{h}\pm |R\rangle_{h})/\sqrt{2}$ , whose electron part has a pronounced bonding nature. The electronic charge distribution of this state is delocalized all over the QDM, as observed in the *X* ^–^ panel of Figure 3b—gray curve. In turn, the first excited state—|*X* ^–^⟩_1_, purple line—places one electron in the antibonding orbital, |σ*⟩_*e*_, thus constituting an electron spin triplet with nonbonding character. Last, the second excited state—|*X* ^–^⟩_2_, green line—places both electrons in |σ*⟩_*e*_ and consequently acquires antibonding character. The electronic charge density in the barrier decreases gradually between such states, as can be observed in Figure 3b.

All in all, we infer from Figure 3 that both *X* ^+^ and *X* ^–^ present a ground state with bonding molecular character, which is sufficiently detached in energy from excited states to concentrate most of the population at room temperature. *X* ^+^ displays the strongest molecular character among excitonic species, as can be observed in the charge density. In turn, *X* ^–^ has the largest hybridization energy, Δ_*X* ^–^_. This is because Δ_X^–^_ is partly set by the tunneling energy and partly by the trion binding energy (see Section S4 in the SI for details). A quantitative estimate of the σ-bond character of *X*, *X* ^+^, and *X* ^–^ is also given (Figure S5), which shows the generality of these findings in QDMs with different *D*_neck_.

The next question to be addressed is the stability of the trion ground state delocalization against possible deviations from the ideal homodimer situation. These can be related to off-centering of the CdSe core,^32^ asymmetries in the crystallographic orientation,^28^ stochastic surface charges,^31^ or fluctuations in the core or shell sizes. The latter is likely to be the most challenging issue. Because the CdSe cores that maximize tunnel coupling are small (typically *r* ≈ 1.2–1.6 nm), minor differences in size between the two cores forming a dimer imply major changes in confinement and Coulomb energies. To look into this problem, we consider the ideal QDM studied so far (core radius *r* = 1.35 nm, shell radius *R* = 3.4 nm, quasi-rod-like neck *n* = 7 nm) and introduce an increasingly heteronuclear character by changing the size of the right core as *r*_r_ = *r* + Δ*r*. Figure 4a shows the evolution of the ground state energy and carrier localization (through the schematic insets) as a function of Δ*r*.

**Figure 4:**
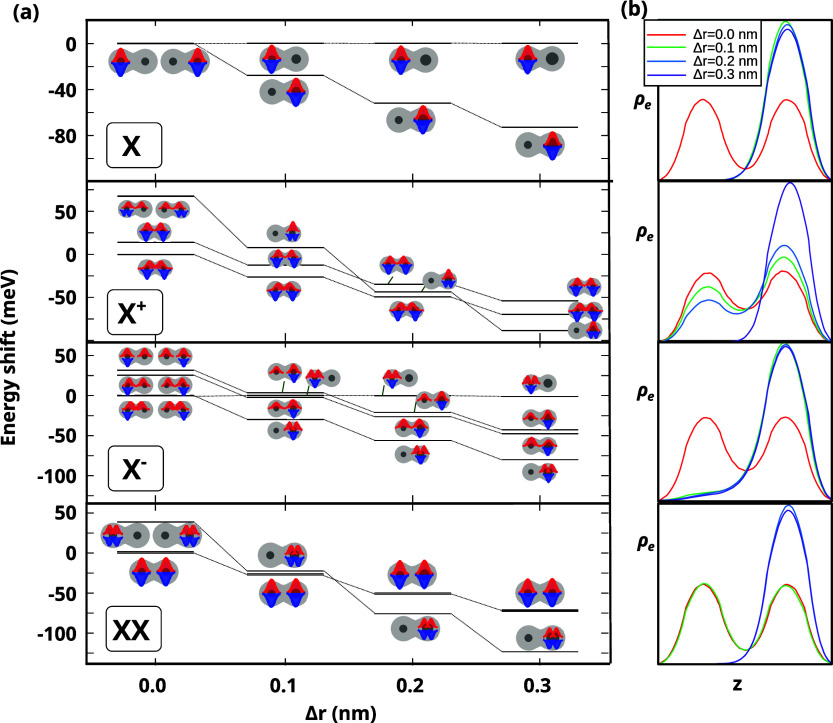
Robustness of the QDM ground state delocalization against core size dispersion for different excitonic species. The left QD has *r*_l_ = 1.35 nm. The right one is *r*_r_ = *r*_l_ + Δ*r*. (a) Energy levels, with schematics of the electron (red) and hole (blue) localization. For each species, the reference energy is that of the ground state in the homodimer limit. (b) Cross-section of the normalized electron charge density along the coupling axis of the QDM for the ground state of each complex and different values of Δ*r*. Notice the stability of *X* ^+^, which preserves delocalization up to Δ*r* = 0.2 nm.

*X* is very fragile. In the homodimer limit, Δ*r* = 0 nm, its ground state shows localized character in either the left or right QD, which are degenerate. However, Δ*r* = 0.1 nm suffices for the right *X* to be 26 meV lower in energy. At Δ*r* = 0.3 nm, the difference rises to 74 meV. This translates into a rapid migration of the electron charge density toward the bigger QD. A quantitative illustration of this process can be found in Figure 4b. When Δ*r* = 0 nm (red line), there is even a probability of finding the electron in either QD, but for Δ*r* ≥ 0.1 nm (other colors), it is almost completely localized in the right QD.

A similar pattern is followed by *XX*, as shown in the bottom panel in Figure 4a. In the homodimer limit, the ground state is composed of one exciton in each QD, but for Δ*r* ≥ 0.2 nm, the weaker confinement of the right QD enables the localized *XX*, with two excitons in the right QD, to be lower in energy. The result is a transfer of electronic charge density toward a single QD, as shown in Figure 4b. This ground state reversal was anticipated by some of us in earlier simulations, and it was consistent with spectroscopic measurements.^26^

A more remarkable behavior is observed for trions. For *X* ^–^, when Δ*r* ≥ 0.1 nm, the kinetic stabilization favors localization in the right QD, similar to the case of *X* and *XX*. Yet, unlike in neutral species, a tail of electron charge density remains in the left QD; see the *X* ^–^ panel in Figure 4b. This is because electron–electron repulsion inside the right QD remains strong, which favors partial delocalization. Even more interesting is the case of *X* ^+^. Here, localizing all carriers inside the bigger QD would require overcoming hole–hole repulsions. These are stronger than electron–electron repulsions because holes are localized inside small CdSe cores. As a consequence, a large change in size (Δ*r* = 0.3 nm) is needed for kinetic stabilization to compensate for such repulsions and localize all the ground state carriers in the right QD; see energy levels in Figure 4a and charge density in Figure 4b. Because CdSe/CdS QDMs have moderate core size dispersion,^22,27^ one can foresee that many of the QDMs will correspond to Δ*r* ≤ 0.2 nm. Hence, when populated with *X* ^+^, they will preserve much of the electronic coupling.

Experimental verification of the electronic coupling of trions in QDMs we predict may be conducted by different means. Magneto-photoluminescence may be used to estimate the electronic charge density in the neck region because the different *g*-factor of core (CdSe) and shell (CdS) implies different Zeeman splitting for localized and delocalized states.^8^ In the specific case of *X* ^–^, delocalization should further enable the formation of a two-electron spin singlet, well separated from triplet states (|*X* ^–^⟩_0_ in Figure 3a). This should remove fine structure details from magneto-luminescence.^33^ Last, direct optical measurement of hybridization energies (Δ_X^+^_ and Δ_e_) is feasible in the trion emission of homodimers and approximate ones in heterodimers. Below, we discuss the case of homodimers. Sections S5 and S6 in the SI give additional data about radiative rates of dimers and heterodimer emission.

Figure 5 shows the calculated spectrum of the prototypical homodimer. In the simulations, we consider all the low-lying states of *X*, *X* ^±^, and *XX* to be equipopulated and disregard nonradiative (Auger) mechanisms. This can be helpful for experimental assignments because in QDMs, the ground states—and some metastable excited states—of trions and biexcitons have been found to be emissive.^26,31^ This behavior is likely connected with the quenched Auger recombination in dimers, which present weaker confinement and greater carrier delocalization than monomers.^22^

**Figure 5:**
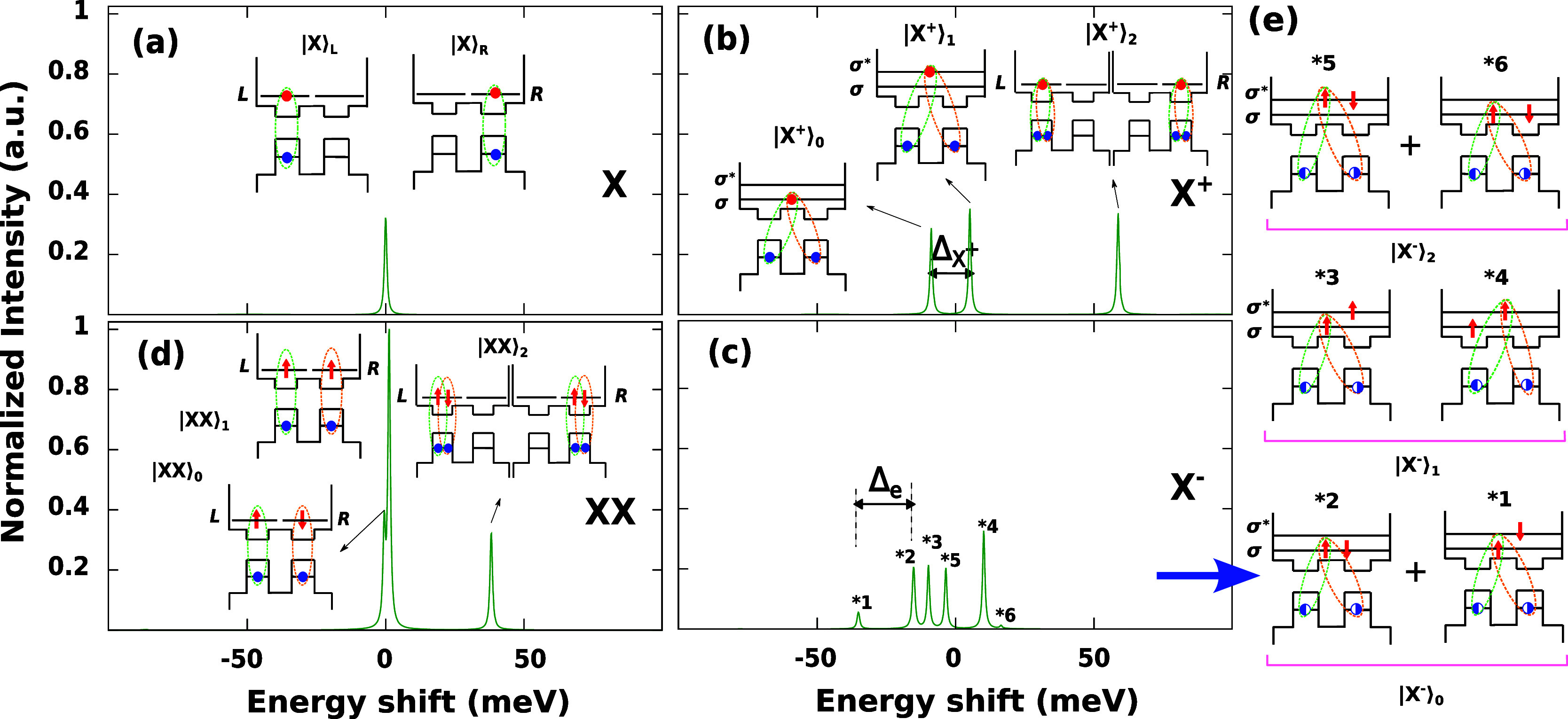
Spectral assignment of the optical interband transitions of *X* (a), *X* ^+^ (b), *X* ^–^ (c), and *XX* (d) in a QDM homodimer with *r* = 1.35 nm, *R* = 3.4 nm, and *n* = 7 nm. The insets show the *e*–*h* recombination process involved in the transition. (e) Recombination processes in the case of *X* ^–^. In the schematics, red and blue circles represent *e* and *h*. Blue semicircles indicate 50% probability of finding the hole in a given dot, i.e., $|h\rangle =(|L\rangle_{h}\pm |R\rangle_{h})/\sqrt{2}$ . In all cases, the reference energy is the emission energy of the fundamental exciton, and the intensity is normalized to that of the highest *XX* peak.

The emission of *X* and *XX* shows no relevant sign of electronic coupling. Figure 5a displays the fundamental *X* transition. A single peak is observed that originates from the recombination of |*X*⟩_0_. As the inset indicates, the peak arises from the equiprobable recombination of *X* in the left or in the right dot, i.e., from |*X*⟩_L_ or |*X*⟩_R_, which are inherent to |*X*⟩_0_. Figure 5d shows the *XX* spectrum. Peaks coming from segregated biexcitons (|*XX*⟩_0_ and |*XX*⟩_1_) are split from that of the localized biexcitons (|*XX*⟩_2_), the energy spacing closely reflecting the *XX* binding energy.

Trions, on the other hand, exhibit clear signs of electronic coupling. Figure 5b shows the emission spectrum of *X* ^+^. In this case, three peaks are observed. From low to high energy, these correspond to the recombination of |*X* ^+^⟩_0_, |*X* ^+^⟩_1_, and |*X* ^+^⟩_2_ states, respectively. The splitting between the two first peaks is a direct measure of the hybridization energy in this system, Δ_*X* ^+^_, as these differ in the occupation of |σ⟩_e_ and |σ*⟩_e_ (see schematic insets). The |*X* ^+^⟩_0_ peak is slightly less intense than that of |*X* ^+^⟩_1_. This is because |*X* ^+^⟩_0_, being a bonding state, deposits some of its electron charge on the QDM barrier, and this reduces the overlap with the holes localized inside the CdSe cores as compared to |*X* ^+^⟩_1_, which is antibonding.

Contrasting the peaks of |*X* ^+^⟩_0_ and |*X* ^+^⟩_1_ with that of |*X* ^+^⟩_2_ brings yet another relevant observation. The two first peaks, which belong to delocalized trions, are strongly red-shifted (∼50 meV) with respect to the third one, which belongs to a localized trion. The original experiments showing red-shifted emission of dimers as compared to monomers contained not only excitons—the only species considered in previous theoretical studies on electronic coupling^29,30,34^ —but also trions, as suggested by the short (Auger-limited) lifetimes.^22^ The experimental redshift may then be partly reflecting the formation of segregated (and possibly delocalized) positive trions.

The emission spectrum of *X* ^–^, shown in Figure 5c, is richer. Several peaks can be observed in this case. A detailed assignment is given in the schematics of Figure 5e. The most significant features are the peaks labeled as 1* and 2*, which originate from the ground state, |*X* ^–^⟩_0_. The main CI configuration of such a state has an electronic part |σ⟩_e1_|σ⟩_e2_. When one of these electrons recombines with a localized hole, transition 2* builds up. Many-body correlations, however, make |*X* ^–^⟩_0_ have secondary configurations with nonbonding character, |σ⟩_e1_|σ*⟩_e2_. When the σ electrons recombine with a hole, transition 1* builds up. Because they come from the same initial (trion) state, the energy shift between peaks 1* and 2* is solely set by the energy difference of the final (|σ*⟩_e_ and |σ⟩_e_) states. Thus, the energy shift provides a direct measurement of the electron hybridization energy, Δ_e_. The neck width dependence of peaks 1* and 2* is shown in Figure S6.

## Conclusions

In conclusion, we have shown that populating QDMs with trions should provide stronger electronic coupling than doing so with excitons or biexcitons, which have gathered the most experimental and theoretical efforts so far. Trion bonding states are sufficiently far in energy from antibonding ones for the molecular coupling to persist up to or near room temperature and are robust against moderate deviations from the homodimer limit, particularly in the case of *X* ^+^. Direct measurement of hybridization energies should be feasible in single-particle emission spectroscopy of trions in homodimer QDMs.

Along with electronic coupling, which is at the base of the entanglement required by different multiqubit architectures,^11,35^ trions combine the strong optical response of excitons with the sensitivity to external fields of free charges, which further makes them appealing for electrical manipulation.^17,32^ These two properties have rendered them useful for the development of quantum information protocols in epitaxial QDMs.^12,13^ The present findings suggest that they may be eventually transferred to colloidal QDMs.

## Supplementary Material



## Notes

Details on the theoretical model; additional calculations of the electronic structure and optical spectra as a function of the QDM shell thickness and neck width; analyses of relative radiative rates for *X*, *X* ^+^, and *X* ^–^; and additional simulations of *X* and *X* ^+^ spectra for heterodimers (PDF)
