# Supplementary material for: Trions Stimulate Electronic Coupling in Colloidal Quantum Dot Molecules
Source: Chem Mater. 2024 Nov 22;36(23):11676–83. doi: 10.1021/acs.chemmater.4c02809 (PMC12315096; doi:10.1021/acs.chemmater.4c02809)
Supplement: Supplementary file 1 [file cm4c02809_si_001.pdf]

Supporting Information for "Trions Stimulate Electronic Coupling in Colloidal Quantum Dot Molecules"

Jordi Llusar† and Juan I. Climente*,‡

†*BCMaterials, Basque Center for Materials, Applications, and Nanostructures, E-48940, Leioa, Spain*

‡*Departament de Química Física i Analítica, Universitat Jaume I, E-12080, Castelló de la Plana, Spain*

E-mail: climente@uji.es

Section S1: Theoretical framework

Calculations of electron, hole, exciton, trion and biexciton states in colloidal QDMs are carried out within k·p theory framework.[1] In second quantization, the Hamiltonian reads:

$$\hat{H} = \sum_{n} \epsilon_n^e \, e_n^+ \, e_n + \sum_{m} \epsilon_m^h \, h_m^+ \, h_m + \frac{1}{2} \sum_{n_i n_j n_k n_l} \langle n_i n_j | \, V^{ee} \, | n_k n_l \rangle \, e_{n_i}^+ e_{n_j}^+ e_{n_k} e_{n_l}$$
$$+ \frac{1}{2} \sum_{m_i m_j m_k m_l} \langle m_i m_j | \, V^{hh} \, | m_k m_l \rangle \, h_{m_i}^+ h_{m_j}^+ h_{m_k} h_{m_l} + \sum_{m_i n_j n_k m_l} \langle m_i n_j | \, V^{eh} \, | n_k m_l \rangle \, h_{m_i}^+ e_{n_j}^+ e_{n_k} h_{m_l}. \tag{1}$$

Here, ϵ_n^e (ϵ_m^h) is the electron (hole) energy in the single-particle state $|n\rangle_e$ ($|m\rangle_h$), e_i and e_i^+ (h_i and h_i^+) are the electron (hole) annihilation and creation operators, and $\langle ij| \, V \, |kl\rangle$ are the two-body Coulomb matrix elements.

To calculate the single-particle (non-interacting) states, we use three-dimensional effective mass (single-band) Hamiltonians for electrons and holes:

$$\hat{H}_e = \frac{\mathbf{p_e}^2}{2\, m_e^*} + E_g + V_e(\mathbf{r_e}), \tag{2}$$

$$\hat{H}_h = \frac{\mathbf{p_{h\perp}}^2}{2\, m_{h\perp}^*} + \frac{\mathbf{p_{h\|}}^2}{2\, m_{h\|}^*} + V_h(\mathbf{r_h}). \tag{3}$$

Here, $\mathbf{p}$ are the momentum operators, $\perp$ and $\|$ label directions perpendicular and parallel to the QDM coupling axis, m^* is the effective mass, E_g the bulk CdSe band gap and $V(\mathbf{r})$ the confining potential. The latter is defined by the conduction (for electrons) and valence (for holes) band offsets between CdSe cores, CdS shell and the organic ligands surrounding the molecule (see Fig. 1 in the main text). Explicit strain and self-energy potentials are disregarded in H_e and H_h for simplicity. The main effect of lattice-mismatch strain in CdSe/CdS quantum dots is to reduce the conduction band offset, from ~ 0.3 eV, to ~ 0.1 eV.[2] This facilitates electron delocalization into the CdS shell, and hence tunneling across the

QDM. For this reason, we use a strained conduction band offset of 0.1 eV in the simulations (see below). The additional strain contribution due to nanocrystal attachment in QDMs is negligible.[3] The choice of a single-band Hamiltonian for holes is justified because CdSe/CdS QDMs have wurtzite crystal structure.[4] Holes are mostly confined in the CdSe core, where crystal field and spin-orbit splittings confer A-band (heavy hole-like) character to the ground state.[5] Coupling to B- and C-bands is expected to become more important away from the band edge region we study, and in systems where the hole (rather than the electron) resonates between the two dots of the QDM.[6]

Material parameters are given in Table 1. We note that the conduction band offset at the CdSe/CdS interface has been debated in the literature, with values generally in the range between 0.0 − 0.3 eV.[7–9] We choose a value of 0.1 eV, which closely corresponds to the strained band offset of CdSe/CdS quantum dots,[2] and gives biexciton binding energies in close agreement with experiments for QDMs.[10] Using higher band offsets will obviously reduce electron tunneling, but it will not change the fact that trion repulsions restore hybridization as compared to excitons.

Table 1: Material parameters used in the calculations. E_g, is taken from Ref.[11] ε for CdSe and CdS are rounded values close to the static dielectric constant.[11] A typical value of organic ligands is used for the environment.[12] All other parameters are taken from Ref.[4] m_0 is the free electron mass and ε_0 the vacuum permittivity

Parameter	CdSe	CdS	Environment	units
m_e	0.112	0.121	1.0	m_0
$m_{h\perp}$	-0.48	-0.376	-1.0	m_0
$m_{h\parallel}$	-1.19	-0.746	-1.0	m_0
V_e	0.0	0.1	5.0	eV
V_h	0.0	-0.64	-5.0	eV
E_g	1.76	–	–	eV
ε	10.0	10.0	2.4	ε_0

As shown in Fig. 1(a) of the main text, the QDM is defined by two spherical CdSe cores with radii r_l and r_r, surrounded by CdS shells. The shells are spherical, with radii R_l and R_r, except in the direction of coupling. Here, the neck growth is simulated following Ref.[7]

Thus, we start with two tangential shell spheres. In the side which connects the two QDs, we convert the half spheres into half ellipsoids so that they overlap each other. We then merge them, and hence, the neck width is dictated by the long axis of the ellipsoid, set by the semi-major axes $n_l = n_r = n$.

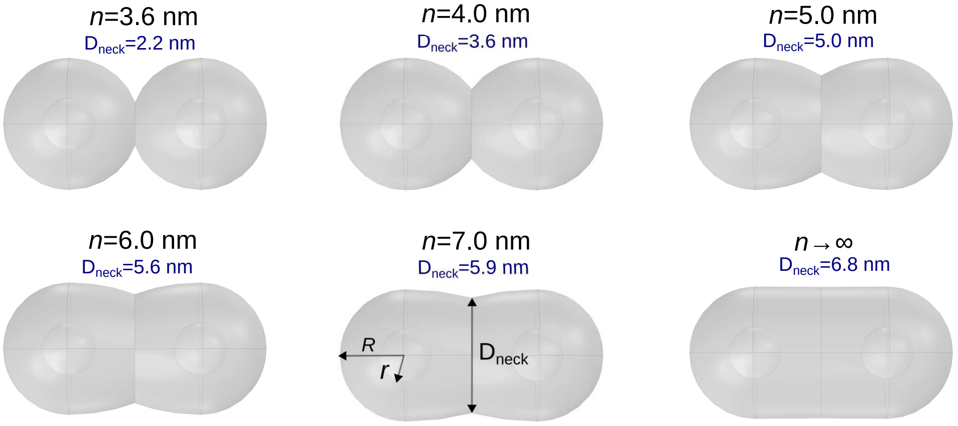

Figure S1: Geometry of QDMs with CdSe core radii $r = 1.35$ nm, CdS shell radii $R = 3.4$ nm and increasing shell ellipsoid semi-axis n. The neck width, D_{neck}, increases with n until it reaches $n = 2R$ (rod limit).

Figure S1 compares the geometry obtained for a QDM with $r_l = r_r = r = 1.35$ nm, $R_l = R_r = R = 3.4$ nm and ellipsoids of increasing eccentricity. One can see that, as n increases, so does the neck width, D_{neck}. In the limit of $n = R$, the dimer is made of two spheres with tangential surface contact. In the limit of $n \to \infty$, the CdS shell reaches a rod shape, which maximizes tunnel-coupling.

Hamiltonians (2) and (3) are integrated numerically using finite elements with Comsol Multiphysics 5.4. The tetrahedral mesh (see Figure S2) introduces tiny asymmetries between left and right sides of the QDM in the direction of coupling. For the mesh size we use, this gives a difference in energy under 1 μeV between left and right QDs. Because the hybridization energy of electrons greatly exceeds this numerical noise, the default states we obtain clearly reflect the symmetric and antisymmetric character of bonding and antibonding states (see $|\sigma\rangle_e$ and $|\sigma^*\rangle_e$ in Fig. 1(b-c) in the main text). For holes, however, the large band offset and heavy mass give negligible hybridization energy. The default hole states we obtain

are then localized in either left or right QD ($|L\rangle_h$ and $|R\rangle_h$ in Fig. 1(b-c) of the main text). In practice, this is not a relevant symmetry breaking: the two states are degenerate to within 1 μeV, such that one can retrieve the symmetric ($|\sigma\rangle_h = 1/\sqrt{2}(|L\rangle_h + |R\rangle_h)$) and antisymmetric ($|\sigma^*\rangle_h = 1/\sqrt{2}(|L\rangle_h - |R\rangle_h)$) eigenstates, with the same energy, through a simple basis rotation.

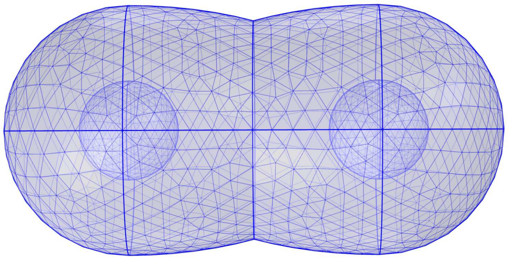

Figure S2: Finite element mesh for the prototypical QDM we study in the main text.

The eigenstates of Hamiltonians (2) and (3) give the orbital (envelope or long range) part of the wave function, $f_n(\mathbf{r_e})$ and $f_m(\mathbf{r_h})$. Complete spinorial wave functions are obtained by including the (short range) Bloch part. For electrons we add the spin:

$$|n\rangle_e = f_n(\mathbf{r_e})\,|s = 1/2, s_z\rangle_n. \tag{4}$$

For holes we add the pseudo-spin (total Bloch angular momentum of A-holes in wurtzite QDs[5,13]):

$$|m\rangle_h = f_m(\mathbf{r_h})\,|j = 3/2, j_z\rangle_m. \tag{5}$$

Because of Kramers degeneracy, electron states with $s_z = \pm 1/2$ (hole states with $j_z = \pm 3/2$) are degenerate. We then build spin-orbitals by duplicating the eigenstates of H_e (H_h), and ascribing different s_z (j_z) projections.

The sums in Hamiltonian (1) run over spin-orbitals, like Eqs.(4) and (5). Thus, Coulomb interactions include both direct and exchange (electron-electron and hole-hole) integrals. Electron-hole exchange terms have negligible influence on the orbital distribution determining the electronic coupling strength, and are then disregarded in this study. Coulomb

interaction matrix elements, $\langle ij|\, V\, |kl\rangle$, are calculated by integrating the Poisson equation in a dielectrically inhomogeneous system, again using Comsol Multiphysics. Boundary conditions are set by defining a large supersphere containing the QDM in the center, and defining monopole potential on its surface. We have checked that higher multipoles have no significant impact on the resulting matrix element. Inside the QDM, we use a unified dielectric constant (see Table 1), neglecting minor differences between CdSe and CdS, as well as anisotropic contributions. The large dielectric contrast between the inorganic QDM and the organic environment leads to strong dielectric confinement effects.[7] These greatly enhance Coulomb interactions inside the QDM as compared to all-inorganic (e.g. epitaxial) QDMs.

Hamiltonian (1) is solved by means of *CItool*, a free-acess full Configuration Interaction code developed by Andrea Bertoni.[14] Fed with the single-particle energies (ϵ_n^e and ϵ_m^h) and Coulomb integrals ($\langle n_i n_j|\, V^{ee}|n_k n_l\rangle$, $\langle m_i m_j|\, V^{hh}|m_k m_l\rangle$, $\langle m_i n_j|\, V^{eh}|n_k m_l\rangle$), the code builds multi-particle states of N^e electrons and N^h holes as a linear combination of Slater determinants:

$$|\Psi_n\rangle = \sum_{l_e=1}^{L_e}\sum_{l_h=1}^{L_h} C^n_{l_e,l_h}|\Phi^e_{l_e}\rangle|\Phi^h_{l_h}\rangle. \tag{6}$$

Here, $|\Psi_n\rangle$ is the n-th multi-particle state, $|\Phi^e_{l_e}\rangle$ and $|\Phi^h_{l_h}\rangle$ are the Slater determinants for electrons and holes, with L_e (L_h) the number of Slater determinants which can be built for N^e electrons (N^h holes) in the basis of spin-orbital states provided. The multi-particle state can be rewritten with a single sum, in order to represent the basis vector of the Hilbert space with a single ket:

$$|\Psi_n\rangle = \sum_{l=1}^{L} C^n_l|\Phi_l\rangle, \tag{7}$$

where L is the dimension of the Hilbert space, i.e. the number of double (electron + hole) Slater determinants considered. Here, $|\Phi_l\rangle = |\Phi^e_{l_e}\rangle|\Phi^h_{l_h}\rangle$, with l_e and l_h fixed by l. CItool projects Hamiltonian (1) onto the above basis set. The Hamiltonian matrix is diagonalized using ARPACK routines.[15] The output of CItool are the eigenenergies (E^n) and coefficients (C^n_l) of the multi-particle states. In our simulations, these are excitons ($N^e = 1, N^h = 1$),

negative trions ($N^e = 2, N^h = 1$), positive trions ($N^e = 1, N^h = 2$) and biexcitons ($N^e = 2, N^h = 2$). We use a basis set formed by the 20 lowest electron spin-orbitals, and the 20 highest hole spin-orbitals. These include the s and p shells of the QDs, as well as the d_{z^2} functions. In the case of electrons, these functions are hybridized along the QDM axis (z in our coordinates frame).

In order to represent the electron or hole charge density of multi-particle states, as in Figs. 2-4 in the main text, we define the electron density operator at a given coordinate point ($\mathbf{r}_e = x$). For single-electron states of the form $|k\rangle_e = f_k(\mathbf{r_e})\,|s = 1/2, s_z\rangle_k$, the operator reads:

$$\hat{\rho}^e(x) = \sum_{k,k'=1}^{K_e} f^*_{k'}(x) f_k(x)\,\delta[(s_z)_{k'} - (s_z)_k]\, e^\dagger_{k'} e_k. \tag{8}$$

Here, K_e is the number of single-electron states in the CI basis set, e_k ($e^\dagger_k$) is the annihilation (creation) operator of an electron in the state $|k\rangle_e$, and the Kronecker delta comes from the orthogonality of Bloch functions. Next, the expectation value is computed:

$$\langle\Psi_n|\hat{\rho}^{(e)}(x)|\Psi_n\rangle = \sum_{l,l'=1}^{L}\left[(C^n_{l'})^* C^n_l\,\delta[l'_h - l_h]\sum_{k,k'=1}^{K_e} f^*_{k'}(x) f_k(x)\,\delta[(s_z)_{k'} - (s_z)_k]\,\langle\Phi^e_{l'_e}|e^\dagger_{k'} e_k|\Phi^e_{l_e}\rangle\right], \tag{9}$$

where $\delta[l'_h - l_h]$ arises from the orthogonality of the hole Slater determinants. Analogous definitions are used for holes.

The output of CItool provides the Slater determinants associated to each coefficient C^n_l using occupation number vectors. This allows us to evaluate matrix elements $\langle\Phi^e_{l'_e}|e^\dagger_{s'} e_s|\Phi^e_{l_e}\rangle$ efficiently by comparing the vectors $e_s|\Phi^e_{l_e}\rangle$ and $e_{s'}|\Phi^e_{l'_e}\rangle$. When identical, the matrix element equals ± 1 (the sign depends on the number of anticommutations involved). When different, the matrix element is zero by orthogonality.

We are interested in the different emission spectra of X, $X^\pm$ and XX in QDMs. Omitting the local field factor arising from the dielectric mismatch,[16] which is the same for all

species in the same QDM, the radiative rate from the initial state, $|\Psi_i^{N_e,N_h}\rangle$ to the final state $|\Psi_f^{N_e-1,N_h-1}\rangle$ is proportional to the electron-hole recombination probability. The emission intensity, calculated within the optical dipole approximation, is then given by:[17]

$$I_{\pm}(h\nu) \propto \sum_i p_i \sum_f |\langle \Psi_f^{N_e-1,N_h-1}|\hat{P}_{\pm}|\Psi_i^{N_e,N_h}\rangle|^2\, \delta(E_i - E_f - h\nu). \tag{10}$$

Here, $h\nu$ is the photon energy, E_f and E_i the energies of final and initial states, p_i is the probability of the initial state being occupied. If thermal equilibrium is assumed, p_i at a given temperature is obtained with Maxwell-Boltzmann statistics. In other instances we assume equipopulation of all states ($p_i = 1$). $\hat{P}_{\pm}$ is the dipole operator describing all possible electron-hole recombinations:

$$\hat{P}_{\pm} = \sum_n^{K_e} \sum_m^{K_h} \langle f_n | f_m \rangle\, \delta[(s_z)_n + (j_z)_m \pm 1]\, e_n\, h_m, \tag{11}$$

where the Kronecker delta grants the (exciton + photon) angular momentum conservation, with the sign depending on the polarization of the incoming light. The dipole matrix elements are evaluated as:

$$\langle \Psi_f^{N_e-1,N_h-1}|\hat{P}_{\pm}|\Psi_i^{N_e,N_h}\rangle = \sum_{l,l'=1}^{L} (C_{l'}^f)^* C_l^i \sum_n^{K_e} \sum_m^{K_h} \langle f_n | f_m \rangle\, \delta[(s_z)_n + (j_z)_m \pm 1] \langle \Phi_{l'} | e_n h_m | \Phi_l \rangle. \tag{12}$$

The integrals $\langle \Phi_{l'} | e_n h_m | \Phi_l \rangle$ are computed by comparing the occupation numbers of $|\Phi_{l'}\rangle$ and $e_n h_m |\Phi_l\rangle$. Again, the outcome is zero when they differ, or ± 1 (depending on the anticommutations) if they coincide. To introduce band broadening in the spectral peaks, the energy conservation function in Eq. (10) is replaced by a Lorentzian curve:

$$\delta(E_i - E_f - h\nu) \rightarrow \frac{1}{1 + ((E_i - E_f - h\nu)/\Gamma)^2}, \tag{13}$$

where Γ is the bandwidth, which we set at 0.5 meV.

Section S2: Effect of shell thickness

In Fig. 2 of the main text we show that the charge density of trions exhibits stronger electronic coupling than that of neutral excitons or biexcitons. Here we show that this result holds for different QDM geometries. Figure S3 shows the electronic charge density in QDMs with the same core radius as in the main text, but varying shell thickness. Thinner shells favor electronic coupling. For a shell with $R = 2.4$ nm (corresponding to a CdS thickness of $R - r = 1.05$ nm), X displays sizable coupling, in agreement with earlier atomistic simulations.[18] In all cases, however, trions display enhanced electronic coupling.

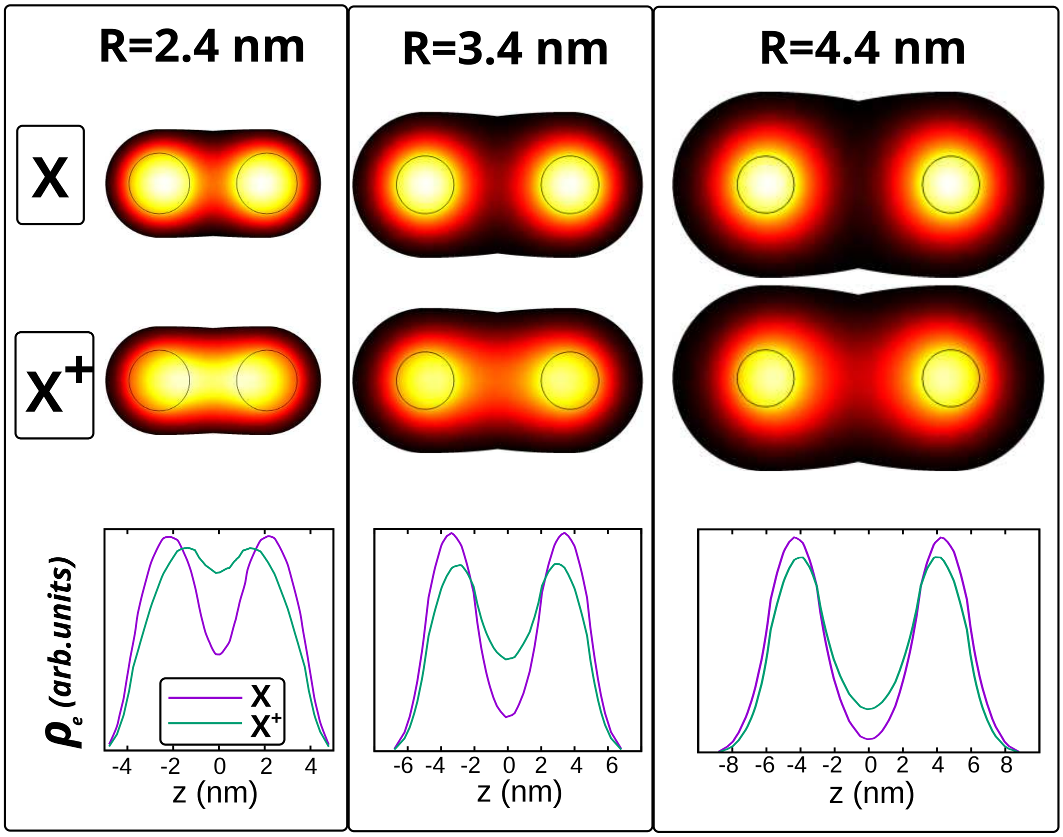

Figure S3: Electronic charge density of excitons and positive trions in QDMs with different shell thickness. In all cases, the core radius is $r = 1.35$ nm, and a rod-shaped CdS shell ($n = 10$ nm) is taken.

Section S3: Molecular and atomic basis set

In Fig. 3 of the main text we analyze the main CI configuration of the lowest states of X, X^+, X^- and XX. As decribed in the previous section, the CI calculations are run on the basis of non-interacting (single-particle) electron and hole states. For electrons, such states are delocalized (see e.g. $|\sigma\rangle_e$ and $|\sigma^*\rangle_e$ in Fig. 1(c)). For holes, they are localized (see e.g. $|L\rangle_h$ and $|R\rangle_h$ in Fig. 1(c)).

For excitons, we find that the lowest states (omitting spin degrees of freedom) have CI expansions of the form:

$$|X\rangle_0^{(1)} = c_1^{(1)} |\sigma\rangle_e |R\rangle_h + c_2^{(1)} |\sigma^*\rangle_e |R\rangle_h + \ldots, \quad (14)$$

$$|X\rangle_0^{(2)} = c_1^{(2)} |\sigma\rangle_e |L\rangle_h + c_2^{(2)} |\sigma^*\rangle_e |L\rangle_h + \ldots, \quad (15)$$

where $c_2^{(1)} \approx -c_1^{(1)}$ and $c_2^{(2)} \approx c_1^{(2)}$, and the ellipsis indicate minor terms of the expansion. These states have a pronounced non-bonding character for electrons (comparable weight of σ and σ^* configurations). We can however rotate from the delocalized (molecular) basis set to a localized (atomic) one. The conversion between the two basis sets is given by the symmetric and antisymmetric linear combinations:

$$|\sigma\rangle_e = (|L\rangle_e + |R\rangle_e)/\sqrt{2}, \quad (16)$$

$$|\sigma^*\rangle_e = (|L\rangle_e - |R\rangle_e)/\sqrt{2}. \quad (17)$$

where $|L\rangle_e$ and $|R\rangle_e$ are electron s-like orbitals localized in left or right QD, respectively. Notice that in Eqs. (16) and (17), for simplicity of the analysis, we have disregarded the influence of the overlap integral ($\langle L|R\rangle$) on the normalization factor. By doing so, the

exciton states can be rewritten as:

$$|X\rangle_0^{(1)} \approx \sqrt{2}c_1^{(1)}\,|R\rangle_e|R\rangle_h, \tag{18}$$

$$|X\rangle_0^{(2)} \approx \sqrt{2}c_1^{(2)}\,|L\rangle_e|L\rangle_h. \tag{19}$$

which reveals that non-bonding states are equivalent to localized states. A similar case holds for XX states. For clarity of exposition, in Fig. 3(a) we use the localized basis set when representing X and XX configurations.

In trions the situation is different, as the majority of states display a dominant (bonding or antibonding) CI configuration. For instance, the lowest states of X^+ (omitting spin degrees of freedom) are of the form:

$$|X^+\rangle_0^{(1)} = c_1^{(1)}\,|\sigma\rangle_e\,(|R\rangle_{h1}|L\rangle_{h2} + |L\rangle_{h1}|R\rangle_{h2}) + \ldots, \tag{20}$$

$$|X^+\rangle_0^{(2)} = c_1^{(2)}\,|\sigma\rangle_e\,(|R\rangle_{h1}|L\rangle_{h2} - |L\rangle_{h1}|R\rangle_{h2}) + \ldots \tag{21}$$

with c_1 much greater than any other coefficient in the expansion. For such states, preserving the delocalized electron basis set is convenient to highlight their molecular nature. We do so in Fig. 3(a).

Section S4: Neck width dependence of hybridization energies

Fig. 3(a) in the main text illustrates the hybridization energies for a homodimer with $n = 7$ nm ($D_{neck} = 5.94$ nm). The hybridization energy of trions ($\Delta_{X^+}, \Delta_{X^-}$) was found to be greater than that of excitons (Δ_X). Here we show that this is systematically true for any neck width.

We define the hybridization energies as the splitting between the lowest state with dom-

inant antibonding character and that with dominant bonding character:

$$\Delta_e = E_{|\sigma^*\rangle_e} - E_{|\sigma\rangle_e}, \tag{22}$$

$$\Delta_X = E_{|X\rangle_0^{(5)-(8)}} - E_{|X\rangle_0^{(1)-(4)}}, \tag{23}$$

$$\Delta_{X^+} = E_{|X^+\rangle_1} - E_{|X^+\rangle_0}, \tag{24}$$

$$\Delta_{X^-} = E_{|X^-\rangle_2} - E_{|X^-\rangle_0}. \tag{25}$$

Here, $|X\rangle_0^{(1)-(4)}$ are the four lowest states of the 8-fold quasi-degenerate exciton ground state multiplet, and $|X\rangle_0^{(5)-(8)}$ are the four highest ones. The rest of states are those defined in Figs. 1 and 3 of the main text. Figure S4(a) represents the hybridization energies as a function of the QDM semi-major axes (n). Figure S4(b) is the same result but as a function of the neck width, $D_{neck} = 2R\sqrt{1-(R/n)^2}$. In all cases the hybridization energy of excitons is negligible compared to that of a non-interacting electron, $\Delta_X \ll \Delta_e$. However, sizable energies are restored for trions ($\Delta_{X^\pm}$).

One can see in Fig. S4 that Δ_{X^+} behaves very similar to Δ_e, even though the magnitude is slightly smaller because the delocalized electron is partly attracted by the holes localized in the cores. By contrast, Δ_{X^-} is greater in magnitude and does not retrieve the limit of zero energy as n (or D_{neck}) decrease. The different behavior of X^+ and X^- is because the states involved in Eqs. (24) and (25) have different nature. As discussed in the previous section, $|X^+\rangle_0$ and $|X^+\rangle_1$ have main CI configurations with clear $|\sigma\rangle_e$ and $|\sigma^*\rangle_e$ components, respectively. Conversely, $|X^-\rangle_0$ and $|X^-\rangle_2$ have mixed electron character, with non-bonding components being non-neglegible. When the CdS neck is wide ($n \geq 6$ nm), it makes sense to describe the states as "mainly bonding" and "mainly antibonding". When the CdSe neck narrows down, however, the weight of non-bonding (localized) components increases and they become dominant. $|X^-\rangle_0$ tends to a localized trion, with the two electrons and the hole in the same QD. $|X^-\rangle_2$ tends to a segregated trion instead, with an exciton in one QD plus another electron in the other QD. Then, Δ_{X^-} is no longer connected to the electron

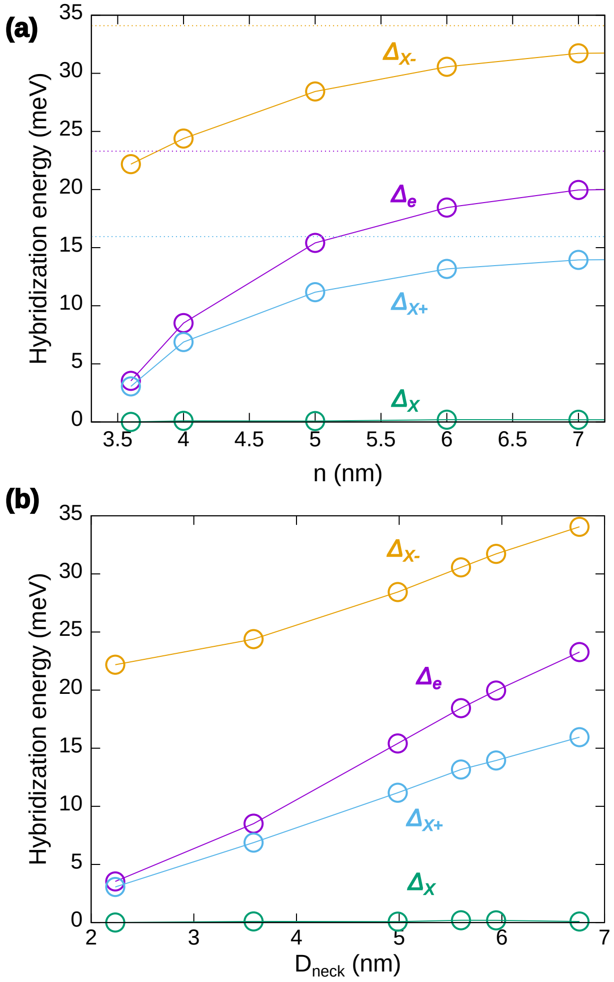

Figure S4: Hybridization energies of exciton and trions in homodimer QDMs, as a function of (a) the semi-major axis, (b) the neck width. The horizontal lines in (a) show the asymptotic limit of $n \to \infty$ for each species. For comparison, the hybridization of a non-interacting electron is shown (Δ_e, purple line). The core and shell dimensions are the same as in the homodimer studied in the main text.

tunnel coupling. Rather, it is connected with the trion binding energy of a monomer. In other words, the value of $\Delta_{X^-} = 22.2$ meV observed for $D_{neck} = 2.2$ in Fig. S4(b) is *mainly* given by the Coulomb stabilization of $|X^-\rangle_0$ as compared to $|X^-\rangle_2$.

In order to quantify the molecular character of a state, we define the following operator:

$$\hat{B_\sigma} = \frac{1}{N_e} \left(\hat{\sigma} + \hat{\sigma^*} \right) . \tag{26}$$

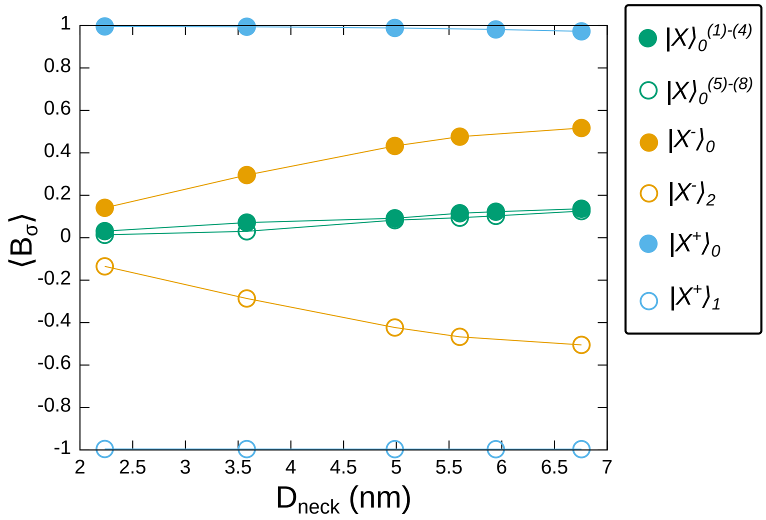

Figure S5: Mean value of the sigma bond character for a homodimer with different neck widths. X states are roughly non-bonding. X^+ states are largely bonding/antibonding. X^- states evolve from non-bonding to moderate bonding or antibonding character as the neck widens.

which gives the strength of the sigma bond as compared to non-interacting electrons. In this expression, N_e is the number of electrons,

$$\hat{\sigma} = (+1)\,(e^{\dagger}_{\sigma\uparrow}\, e_{\sigma\uparrow} + e^{\dagger}_{\sigma\downarrow}\, e_{\sigma\downarrow}) \tag{27}$$

and

$$\hat{\sigma}^{*} = (-1)\,(e^{\dagger}_{\sigma^{*}\uparrow}\, e_{\sigma^{*}\uparrow} + e^{\dagger}_{\sigma^{*}\downarrow}\, e_{\sigma^{*}\downarrow}). \tag{28}$$

When applied on a state $|\Psi_n\rangle$, the creation operator $e^{\dagger}_{\sigma^{*}\uparrow}$ occupies an electron spin-orbital with envelope function $|f\rangle = |\sigma^{*}\rangle_e$ and spin part $|s = 1/2, s_z = +1/2\rangle$ on every Slater determinant $|\Phi^{e}_{l_e}\rangle$ in Eq. (6). The rest of operators in Eqs. (27) and (28) behave alike. In the simple case of single-electron states, one can easily determine the mean values of $\hat{B}_{\sigma}$ for

a few representative states:

$$|\sigma\rangle_e|s,s_z\rangle \rightarrow \langle\hat{B}_\sigma\rangle = +1, \tag{29}$$

$$|\sigma^*\rangle_e|s,s_z\rangle \rightarrow \langle\hat{B}_\sigma\rangle = -1, \tag{30}$$

$$|L\rangle_e|s,s_z\rangle \rightarrow \langle\hat{B}_\sigma\rangle = 0, \tag{31}$$

$$|R\rangle_e|s,s_z\rangle \rightarrow \langle\hat{B}_\sigma\rangle = 0. \tag{32}$$

These values correspond to pure bonding, pure antibonding, and pure non-bonding states. In multi-particle states the molecular character is in between the limit cases above. Figure S5 shows the expectation value $\langle\hat{B}_\sigma\rangle$ calculated for low-lying exciton and trion states as a function of the QDM neck width. All X states are roughly non-bonding, as $\langle\hat{B}_\sigma\rangle \approx 0$. This is consistent with their largely localized nature. $|X^+\rangle_0$ ($|X^+\rangle_1$) is largely bonding (antibonding), as $\langle\hat{B}_\sigma\rangle \approx 1$ ($\langle\hat{B}_\sigma\rangle \approx -1$). This is in line with the strong molecular character observed in the charge density (see main text). One should however bear in mind that $\langle\hat{B}_\sigma\rangle \approx \pm 1$ means as bonding (antibonding) as a single-electron state. For narrow necks, non-interacting electrons have weak tunneling, so $\langle\hat{B}_\sigma\rangle \approx \pm 1$ does not imply large hybridization energies. Last, $|X^-\rangle_0$ and $|X^-\rangle_2$ evolve from non-bonding to moderate bonding and antibonding character, respectively, as the QDM neck widens.

A closer look into the hybridization energy Δ_e, as revealed by X^- emission, is given in Figure S6. As mentioned in the main text, in a homodimer the correlated state $|X^-\rangle_0$ is formed by a combination of CI configurations such as those depicted in Fig. S6(a). Namely, a main configuration with two bonding electrons, $|\sigma\rangle_e\,|\sigma\rangle_e$, another with non-bonding character, $|\sigma\rangle_e\,|\sigma^*\rangle_e$, plus other minor configurations. The e-h recombination in the first configuration leaves a $|\sigma\rangle_e$ electron behind, producing a peak which we label as 2^*. The recombination in the second configuration gives rise to an additional peak (this is known as a shake-up process[19]), whose final state is $|\sigma^*\rangle_e$ and we label as 1^*. The splitting between 1^* and 2^* reflects precisely the single-electron hybridization energy, Δ_e. When the QDM neck width

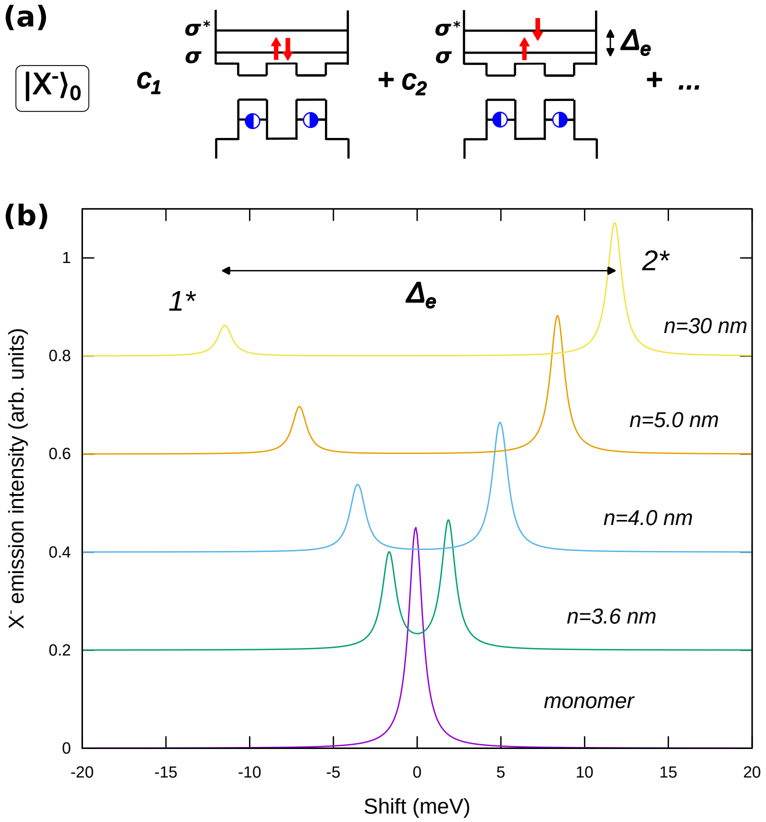

Figure S6: (a) Dominant CI configurations for the trion ground state, $|X^-\rangle_0$. The coefficients depend on the neck width (see text), but in general $|c_1| \gtrsim |c_2|$. (b) Emission spectrum of X^- calculated at T=20 K, for a monomer (bottom curve) and homodimers of increasing neck width (top curves). The spacing between the two peaks reflects the single-electron hybridization energy, Δ_e.

is small, tunneling (Δ_e) is quenched. The result are two configurations having comparable weight ($c_1^2 \approx c_2^2$ in the expansion). Then, as shown in Fig. S6(b) for $n = 3.6$ nm, the emission of $|X^-\rangle_0$ displays two nearby peaks (1^* and 2^*) of comparable intensity. With increasing neck width, however, Δ_e increases.[3,20] This makes the peaks split farther apart. At the same time, placing antibonding orbitals higher in energy reduces the weight of c_2. Consequently, peak 1^* (the shake-up peak) loses intensity.

Section S5: Radiative rates

Here we compare the relative radiative rate of X, X^+ and X^- in homodimer QDMs and that of monomers (QDs). Normalized rates are obtained as the intensities calculated with Eq. (10), referred to that of the bright X in the monomer. A temperature of $T = 20$ K is taken, such that only the ground state of the initial species ($|X\rangle_0$, $|X^+\rangle_0$, $|X^-\rangle_0$) has significant thermal population p_i.

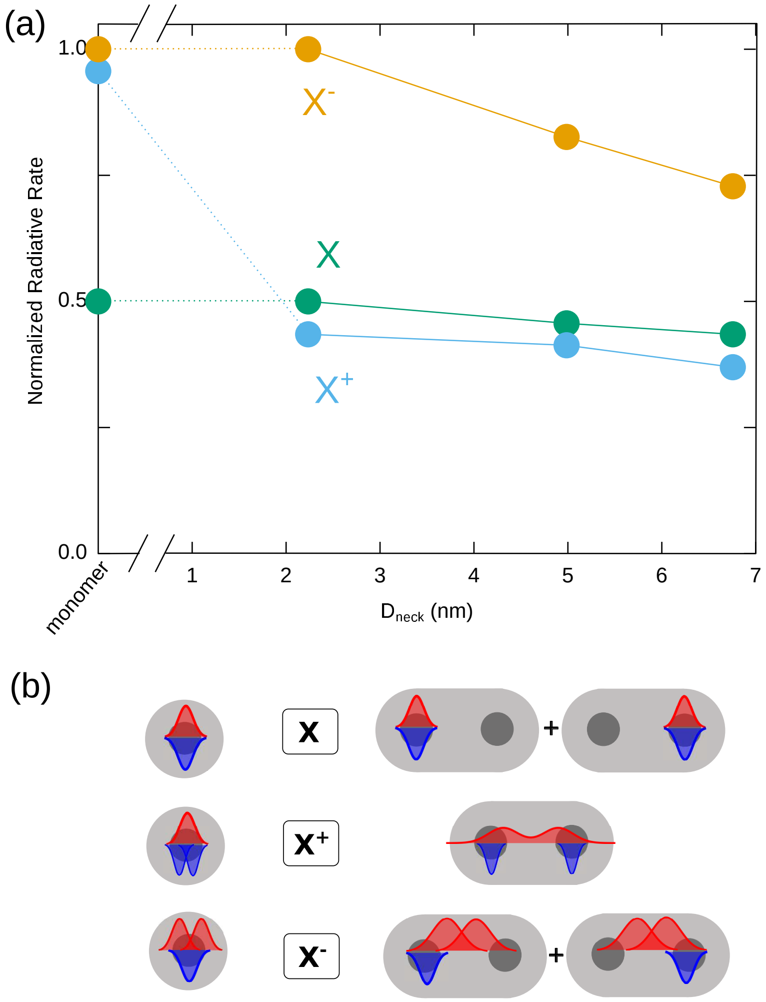

Figure S7: (a) Radiative rate of X, X^+ and X^- in a monomer (left) and dimer with increasing neck width. All the rates are normalized to that of the bright exciton in a monomer. (b) Schematic of the charge distribution in the monomer (left) and rod-like dimer (right) limits. In the dimer, localized charges can be in left or right dot indistinctly.

Figure S7(a) shows the radiative rate of monomers (left-most dot) and dimers (rest of

dots) with increasing neck width. While the radiative rate of excitons (green dots) is similar in dimers and monomer, significant differences arise in the case of trions (blue and orange dots). These can be rationalized in terms of spin selection rules and e-h overlap. Below we explain the trends in detail.

In monomers, the emission rate of trions is about twice that of excitons. This is a well-known result, which follows from spin selection rules in the e-h recombination process. For X, the electron spin has angular momentum $s = 1/2$ and the hole Bloch angular momentum ($j = 3/2$). This gives excitons with total angular momentum $F_i = s \otimes j = 1 + 2$. Because the final state after recombination (closed shell state) has $F_f = 0$, only half of the exciton states (bright excitons, $F_i = 1$) can recombine radiatively in single-photon processes. The X normalized rate is then ~ 0.5. For X^-, the two-electron ground state forms a spin-singlet ($S = 0$), so that the total angular momentum is $F_i = 0 \otimes 3/2 = 3/2$, which can recombine to a final electron state (s_f=1/2) with no restriction. The normalized rate is then about twice that of X. Analogous reasoning holds for X^+, where the two holes confined in the core form a pseudo-singlet. Small deviation from the relative ratio 0.5:1:1 for X:X^+:X^- are found in our simulations. These arise from minor differences in the e-h overlap in each species.

In dimers, excitons have similar rates as in monomers, see green dots in Fig. S7(a). This is because X localize in either left or right QD, which is reminiscent of monomers, see X schematic in Fig. S7(b), Trions, on the other hand, have different behavior. In the case of X^+, the normalized rate changes from ~ 1.0 to ~ 0.5 when switching from monomer to dimer. That is, the radiative rate becomes comparable to that of excitons. The reason is that the two holes localize in opposite cores (see schematic in Fig. S7(b)). Their mutual interaction is then so weak, that they can be considered as two isolated pseudo-spin doublets. The electron can recombine with either left or right hole, with the other hole being essentially a spectator. The selection rules are then the same as for X. In the case of X^-, the two electrons occupy different QDs but their electronic coupling is sizable. This consitutes a fundamental difference. Their mutual interaction enables the formation of a spin singlet, and hence the

preservation of the selection rules observed in monomers. In fact, the normalized rate for narrow necks is ~ 1.0. With increasing neck width, the radiative rate decreases because an increasing amount of electron charge density is deposited in the neck region. This reduces the e-h overlap.

Section S6: Positive trion emission in heterodimers

In Fig. 5 of the main text we provide a detailed assignment of the emission spectrum of homodimer QDMs. Here we completement that result with the emission spectrum of heterodimers. We focus on the case of X^+, because its electronic coupling is relatively robust against deviations from the homodimer geometry (Fig.4). The X case is also shown as a reference. The spectra of both species are plotted in Figure S8. The bottom curves show the spectrum of the homodimer QDM ($r = 1.35$ nm, $R = 3.4$ nm, $n = 7$ nm). As one moves towards the top, the spectra correspond to more asymmetric QDMs, with one of the two cores increasing its radius by Δr (say, $r_r = r_l + \Delta_r$). In all cases, the reference energy is the emission energy of the fundamental exciton of the homodimer. To account for possible meta-stable, emissive states, we consider equiprobable occupation of all X and X^+ states.

The exciton spectrum, Fig. S8(a), is simple. The emission peak of $|X\rangle_0$ splits into two with increasing Δr. One peak redshifts and the other stays at the same energy. These correspond to $|X_R\rangle$ and $|X_L\rangle$, respectively (assuming the right QD increases in size). For X^+, the peaks also split into two with Δr. The lines show the evolution for $|X^+\rangle_0$ (dotted lines), $|X^+\rangle_1$ (dashed lines) and $|X^+\rangle_2$ (dotted-dashed lines). A few additional peaks build up, which arise from p-hole states in the bigger core. These are unlikely to be observed in experiments, as they will likely decay non-radiatively to the lowest (s-hole) orbital of the QD.

It is worth noting that the lowest peaks in the X^+ spectrum correspond to trions with one hole in each QD, even when $\Delta r = 0.3$ nm. This is in spite of the X^+ ground state

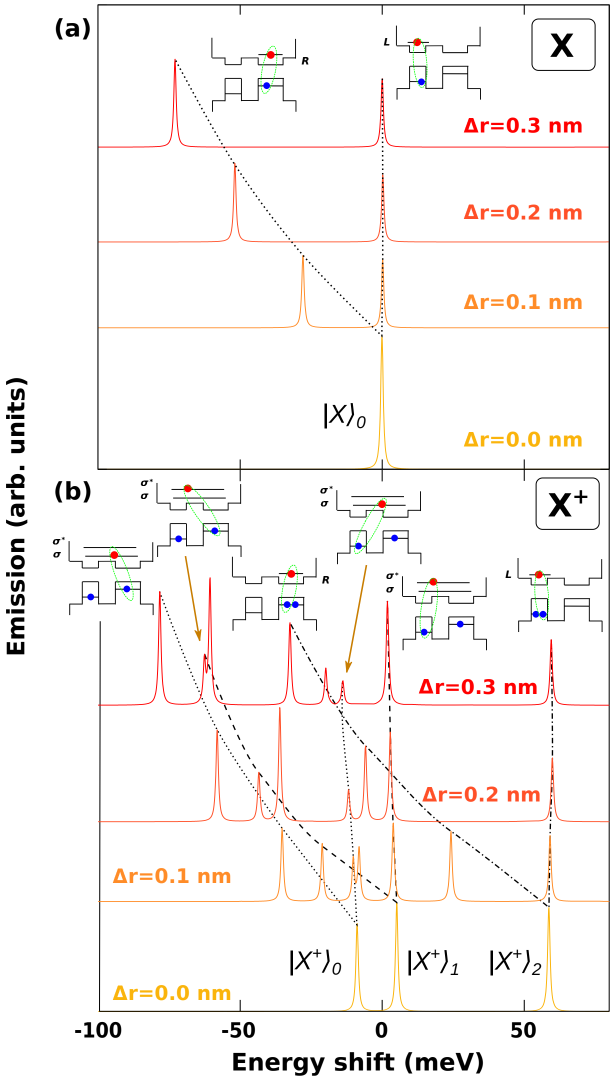

Figure S8: Low energy interband transitions of homo and heterodimer QDMs for (a) exciton and (b) positive trion. $\Delta r = r_l - r_r$ is the difference in size between the two cores. The rest of QDM dimensions are the same as in Fig.5 of the main text. Lines are guides to the eyes showing the evolution of the peaks with increasing asymmetry. The insets illustrate schematically the particles recombining for each transition.

being that with two holes in the same QD (see $\Delta r = 0.3$ nm in Fig.4 of the main text). The energetic ordering in the emission spectrum reflects the difference in energy between initial (trion) and final (hole) state. This amount can be roughly approximated as the energy of the recombining e-h pair within the trion complex. In all the initial states under consideration ($|X^+\rangle_0$ to $|X^+\rangle_2$), the e can recombine with a h in left or right QDs. If the recombining e-h pair is in the left QD (which does not change size with Δr), its energy is about the same

as in the homodimer. If the recombining *e*-*h* pair is in the right QD (which stabilizes with Δr), its energy decreases. The stabilization of the *e*-*h* pair in the right dot is comparable for all the transitions, so the segregated trion emission remains as the lowest in energy.

References

(1) Voon, L. C. L. Y.; Willatzen, M. *The kp method: electronic properties of semiconductors*; Springer Science & Business Media, 2009.

(2) Segarra, C.; Climente, J. I.; Polovitsyn, A.; Rajadell, F.; Moreels, I.; Planelles, J. Piezoelectric control of the exciton wave function in colloidal CdSe/CdS nanocrystals. *The Journal of Physical Chemistry Letters* **2016**, *7*, 2182–2188.

(3) Verbitsky, L.; Jasrasaria, D.; Banin, U.; Rabani, E. Hybridization and deconfinement in colloidal quantum dot molecules. *The Journal of Chemical Physics* **2022**, *157*, 134502.

(4) Cui, J.; Panfil, Y. E.; Koley, S.; Shamalia, D.; Waiskopf, N.; Remennik, S.; Popov, I.; Oded, M.; Banin, U. Colloidal quantum dot molecules manifesting quantum coupling at room temperature. *Nature Communications* **2019**, *10*, 5401.

(5) Planelles, J.; Rajadell, F.; Climente, J. I. Hole band mixing in CdS and CdSe quantum dots and quantum rods. *The Journal of Physical Chemistry C* **2010**, *114*, 8337–8342.

(6) Doty, M.; Climente, J.; Korkusinski, M.; Scheibner, M.; Bracker, A.; Hawrylak, P.; Gammon, D. Antibonding ground states in InAs quantum-dot molecules. *Physical review letters* **2009**, *102*, 047401.

(7) Panfil, Y. E.; Shamalia, D.; Cui, J.; Koley, S.; Banin, U. Electronic coupling in colloidal quantum dot molecules; the case of CdSe/CdS core/shell homodimers. *The Journal of Chemical Physics* **2019**, *151*, 224501.

(8) Rainò, G.; Stöferle, T.; Moreels, I.; Gomes, R.; Kamal, J. S.; Hens, Z.; Mahrt, R. F. Probing the wave function delocalization in CdSe/CdS dot-in-rod nanocrystals by time- and temperature-resolved spectroscopy. *ACS Nano* **2011**, *5*, 4031–4036.

(9) Müller, J.; Lupton, J.; Lagoudakis, P.; Schindler, F.; Koeppe, R.; Rogach, A.; Feldmann, J.; Talapin, D.; Weller, H. Wave function engineering in elongated semiconductor nanocrystals with heterogeneous carrier confinement. *Nano letters* **2005**, *5*, 2044–2049.

(10) Frenkel, N.; Scharf, E.; Lubin, G.; Levi, A.; Panfil, Y. E.; Ossia, Y.; Planelles, J.; Climente, J. I.; Banin, U.; Oron, D. Two Biexciton Types Coexisting in Coupled Quantum Dot Molecules. *ACS Nano* **2023**, *17*, 14990–15000.

(11) Adachi, S. *Handbook on physical properties of semiconductors: II-VI semiconductors*; Springer Science & Business Media, 2004; Vol. 3.

(12) de Sousa, F. F.; Moreira, S. G.; dos Santos, d. S.; Shirsley, J.; Nero, J. D.; Alcantara Jr, P. Dielectric properties of oleic acid in liquid phase. *Journal of Bionanoscience* **2009**, *3*, 139–142.

(13) Chuang, S.; Chang, C. k. k·p method for strained wurtzite semiconductors. *Physical Review B* **1996**, *54*, 2491.

(14) Bertoni, A. CItool. `https://github.com/andreabertoni/citool`, 2011; [Online; accessed 26-August-2024].

(15) Lehoucq, R. B.; Sorensen, D. C.; Yang, C. *ARPACK users' guide: solution of large-scale eigenvalue problems with implicitly restarted Arnoldi methods*; SIAM, 1998.

(16) Lavallard, P. Light emission by semiconductor nanostructures in dielectric medium or close to plane interface. *Acta Physica Polonica A* **1996**, *90*, 645–666.

(17) Jacak, L.; Hawrylak, P.; Wojs, A. *Quantum dots*; Springer Science & Business Media, 2013.

(18) Levi, A.; Hou, B.; Alon, O.; Ossia, Y.; Verbitsky, L.; Remennik, S.; Rabani, E.; Banin, U. The Effect of Monomer Size on Fusion and Coupling in Colloidal Quantum Dot Molecules. *Nano Letters* **2023**, *23*, 11307–11313.

(19) Llusar, J.; Climente, J. I. Nature and control of shakeup processes in colloidal nanoplatelets. *ACS Photonics* **2020**, *7*, 3086–3095.

(20) Cui, J.; Koley, S.; Panfil, Y. E.; Levi, A.; Ossia, Y.; Waiskopf, N.; Remennik, S.; Oded, M.; Banin, U. Neck barrier engineering in quantum dot dimer molecules via intraparticle ripening. *Journal of the American Chemical Society* **2021**, *143*, 19816–19823.
